# Planning a sports training program using Adaptive Particle Swarm Optimization with emphasis on physiological constraints

**DOI:** 10.1186/s13104-017-3120-9

**Published:** 2018-01-08

**Authors:** Nattapon Kumyaito, Preecha Yupapin, Kreangsak Tamee

**Affiliations:** 1 Department of Computer Science and Information Technology, Faculty of Science, Naresuan University, Phitsanulok, 65000 Thailand; 2 Research Center for Academic Excellence in Nonlinear Analysis and Optimization, Faculty of Science, Naresuan University, Phitsanulok, 65000 Thailand; 3 Computational Optics Research Group, Advanced Institute of Materials Science, Ton Duc Thang University, District 7, Ho Chi Minh City, 700000 Vietnam; 4 Faculty of Electrical & Electronics Engineering, Ton Duc Thang University, District 7, Ho Chi Minh City, 700000 Vietnam

**Keywords:** Sports training plan, Training-performance interaction models, Physiological constraints, Particle Swarm Optimization

## Abstract

**Objective:**

An effective training plan is an important factor in sports training to enhance athletic performance. A poorly considered training plan may result in injury to the athlete, and overtraining. Good training plans normally require expert input, which may have a cost too great for many athletes, particularly amateur athletes. The objectives of this research were to create a practical cycling training plan that substantially improves athletic performance while satisfying essential physiological constraints. Adaptive Particle Swarm Optimization using *ɛ*-constraint methods were used to formulate such a plan and simulate the likely performance outcomes. The physiological constraints considered in this study were monotony, chronic training load ramp rate and daily training impulse.

**Results:**

A comparison of results from our simulations against a training plan from British Cycling, which we used as our standard, showed that our training plan outperformed the benchmark in terms of both athletic performance and satisfying all physiological constraints.

**Electronic supplementary material:**

The online version of this article (10.1186/s13104-017-3120-9) contains supplementary material, which is available to authorized users.

## Introduction

Sports training is a process intended to improve athletic performance by means of developing both the physical and mental conditions of the athlete. Sports training can, however, have an opposite and detrimental effect to that intended. A positive result would be an improvement of physical fitness while a negative result would be an increase in fatigue. Training-performance interaction models [1, 2] have defined the relationship between sports training programs and the intended results. Successful athletes utilize a training-performance interaction model to plan wisely in advance of training and rest at appropriate intervals to maximize physical fitness improvement while minimizing the chronic fatigue. While high performance is the ultimate goal of an athlete, detrimental outcomes affecting athletic performance are likely when physiological constraints are not considered, resulting in the risk of overtraining. These physical constraints include training monotony [3], and chronic training load (CTL) ramp rate [4]. While there are a number of apps available [5, 6] their cost is substantial, but there is little evidence to suggest that their use enables a substantial rise of athletic performance. Some research has been undertaken on the generation of sports training plans simply, quickly and efficiently [7–11]. However, while these ‘simple’ training plans have been shown to improve athletic performance, the systems are somewhat impractical with no mechanisms to handle or manipulate necessary constraints.

Most of this related work discusses the search for a global optimal solution to scheduling sports training programs. According to [12], particle swarm optimization (PSO) is the most prevalent swarm intelligence-based optimization algorithm. This algorithm has significant advantages over previous optimization schemes [13] and has been successfully extended to constrained optimization [14]. However, there is little previous research to be found that applies PSO to the construction of optimal training programs.

In [15] a modified form of PSO that applies *ɛ* constraint methods, referred to as adaptive PSO, was used to generate an optimal cycling training plan using simulated athlete data. The result is a cycling training plan purposed to enhance athletic performance by taking into account physiological constraints such as training monotony [3], CTL ramp rate [4] and daily training load. The latter physiological constraint was derived from the British Cycling’s training plan for inclusion in our research.

## Main text

### Problem formulation

This section defines a training plan for a simulated athlete. An 8-week training plan is considered as appropriate preparation for endurance sports [16]. The training plan consists of 56 training sessions each of which introduces a daily training goal by means of average heart rate (HR) in units of beats per minute (bpm) and activity duration (D) in minutes (min).

The boundary of the HR data was extracted from the simulated athlete, 35 years old male who has 51 bpm at resting state, 165 bpm as FTHR and 189 bpm as maximum HR. The lower bound is resting HR and the upper bound is the maximum HR. For ease of use, the training plan should be personalized to the individual athlete. Functional Threshold Heart Rate (FTHR) is considered as a factor that reflects the current level of the athlete’s physical fitness. The Coggan’s training zone [17] corresponding to the simulated athlete’s FTHR is adopted. The boundary of training duration was observed from the training behavior of national athletes which ranged between 30 min and 5 h. The classified heart rate training zones and duration of training zones are illustrated in Table 1.

**Table 1:** Heart rate zone and duration zone

HR zone	HR (bpm)	HR (% of FTHR)	Duration zone	Duration (min)
0	51–81	30.91–49.09	0	30
1	82–112	49.7–67.88	1	60
2	113–124	68.48–75.15	2	90
3	137–146	75.76–82.42	3	120
4	137–146	83.03–88.48	4	150
5	147–155	89.09–93.94	5	180
6	156–165	94.55–100.00	6	210
7	166–173	100.61–104.85	7	240
8	174–181	105.45–109.70	8	270
9	182–189	110.30–114.55	9	300

### Particles encoding

The PSO technique begins by randomly initiating the number of potential training plans as a collection of particles or a swarm. Each particle is encoded into a 112-dimension array from a given training plan of 56 sessions. Each training session has HR and duration data. Thus, at iteration *r,* the *i*th solution that includes *M* training sessions can be expressed as

$$T_{i}^{r} = \left\{ {HR_{i,1}^{r} ,D_{i,1}^{r} ,HR_{i,2}^{r} ,D_{i,2}^{r} HR_{i,3}^{r} ,D_{i,3}^{r} , \ldots ,HR_{i,M}^{r} ,D_{i,M}^{r} } \right\}$$

A full codification of a particle can then be written as

$$HR_{1} D_{1} \,HR_{2} D_{2} \,HR_{3} D_{3} \ldots HR_{56} D_{56}$$

### Objective function

All potential solutions represented by particles are evaluated by Banister’s model which can be stated as Eq. 1.

1 $$p_{t} = p_{0} + \left( {k_{1} \sum\limits_{i = 0}^{t - 1} {w_{i} e^{{ - (t - i)/r_{1} }} } } \right) - \left( {k_{2} \sum\limits_{i = 0}^{t - 1} {w_{i} e^{{ - (t - i)/r_{2} }} } } \right)$$

When athletes train over a certain period of time, they both gain fitness and become fatigued as the positive and negative outcomes. Athletic performance (*p*_*t*_) is the summation of basic athletic performance *p*_0_, the fitness $\left( {k_{1} \sum\nolimits_{i = 0}^{t - 1} {w_{i} e^{{ - (t - i)/r_{1} }} } } \right)$ gained and the fatigue $\left( {k_{2} \sum\nolimits_{i = 0}^{t - 1} {w_{i} e^{{ - (t - i)/r_{2} }} } } \right)$ ‘accumulated’ from training for *t* days. However, the amplitude of fatigue gain (*k*_2_) and the fatigue decay rate (*r*_2_) is higher than fitness gain (*k*_1_) and fitness decay rate (*r*_1_). In this study, all model parameters were defined by the results of the model fitting from Busso et al. [1].

The training load *w*_*i*_ for the *i*th training session can be obtained by Banister’s Training IMPulse (TRIMP) model which can be formulated as Eq. 2.

2 $$w_{i} (d_{i} ,\overline{{hr_{i} }} ) = d_{i} \cdot {}^{norm}HR(\overline{{hr_{i} }} ) \cdot e^{{y \cdot {}^{norm}HR(\overline{{hr_{i} }} )}}$$

*d*_*i*_ is the duration in minutes of a training session on the *i*th day, *y* is the model constant (1.92 for males and 1.67 for females [2]), $\overline{{hr_{i} }}$ is the average heart rate throughout a training session on the *i*th day, and ^*norm*^*HR*() is the normalized $\overline{{hr_{i} }}$ throughout a training session on the *i*th day, which is determined by Eq. 3

3 $${}^{norm}HR(\overline{{hr_{i} }} ) = {{\left( {\overline{{hr_{i} }} - {}^{resting}HR} \right)} \mathord{\left/ {\vphantom {{\left( {\overline{{hr_{i} }} - {}^{resting}HR} \right)} {\left( {{}^{\hbox{max} }HR - {}^{resting}HR} \right)}}} \right. \kern-0pt} {\left( {{}^{\hbox{max} }HR - {}^{resting}HR} \right)}}$$

where $\overline{{hr_{i} }}$ is the average heart rate during a training session the *i*th day. ^*resting*^*HR* is the athlete heart rate at resting state, and ^max^*HR* is the athlete maximum heart rate.

### Physiological constraints

Due to minimizing the risk of overtraining and injuries, the sports training plan need to be satisfied by related physiological constraints. In this paper, 3 physiological constraints of cycling training domain are determined as follow: training monotony, CTL ramp rate and daily TRIMP.

Training monotony is a factor of training with a monotonous pattern may consequence becoming overtrained, which described in [3]. The CTL ramp rate [4] was used as the progressive increase restriction of training load so that athletes can avoid being overtrained. The last introduced constraint is the daily training load limitation. This constraint aims to eliminate excessive training sessions. From investigating on the standard training plan from British Cycling [18], this study determined 450 of TRIMP as the maximum daily training load.

All of these constraints can be considered as inequality constraints, and when applied indicate a training monotony value in a training plan should not be over 1.5, The CTL ramp rate score should be under 5 for < 4 weeks, and the daily TRIMP should be kept lower than 450. Equality constraints are not presented in this study.

### Constrained optimization

The Adaptive PSO algorithm, using the *ɛ*-constrained method [15] separately uses the particle objective and constraint violation values to determine which particle is the better. We adopted the methods in [15] that limit the particle maximum velocity adaptively to decrease the possibility of flying over a feasible region as a pseudocode shows this in more detail (see Additional file 1).

### Procedure

We modified the source code of Pyswarm [19] and represent the new algorithm as pseudocode shown above. The parameters for the *ɛ* constrained method were defined by the constraint violation being given by the square sum of all constraints (*p* = 2). The *ɛ*-level is assigned to 0 which means that the problems are solved in lexicographic order where the constraint violations precede the objective function. The number of groups *N*_*g*_ = 4, the number of particles in a group *n*_*g*_ = 25, the weight of the number of the currently feasible particle is *F*_*λ*_ = 0.2, the threshold of updating *F*_*θ*_ = 0.05. The parameters for PSO are defined as follows: the number of particles *N* = 100 (= 5 × 25), *w*^0^ = 0.9, *w*^*T*^ = 0.4, the initial velocity is 0, and the maximum velocity $V_{{MAX_{j} }}$ is adaptively controlled. The maximum number of iterations is 5000 (50,000 fitness evaluations). Independent runs were performed 30 times.

## Discussion

We select and analyze the run with the best athletic performance. The discussion of our results is discussed in terms of training patterns, athletic performance, and constraints handling.

### Training patterns

**Fig. 1:**
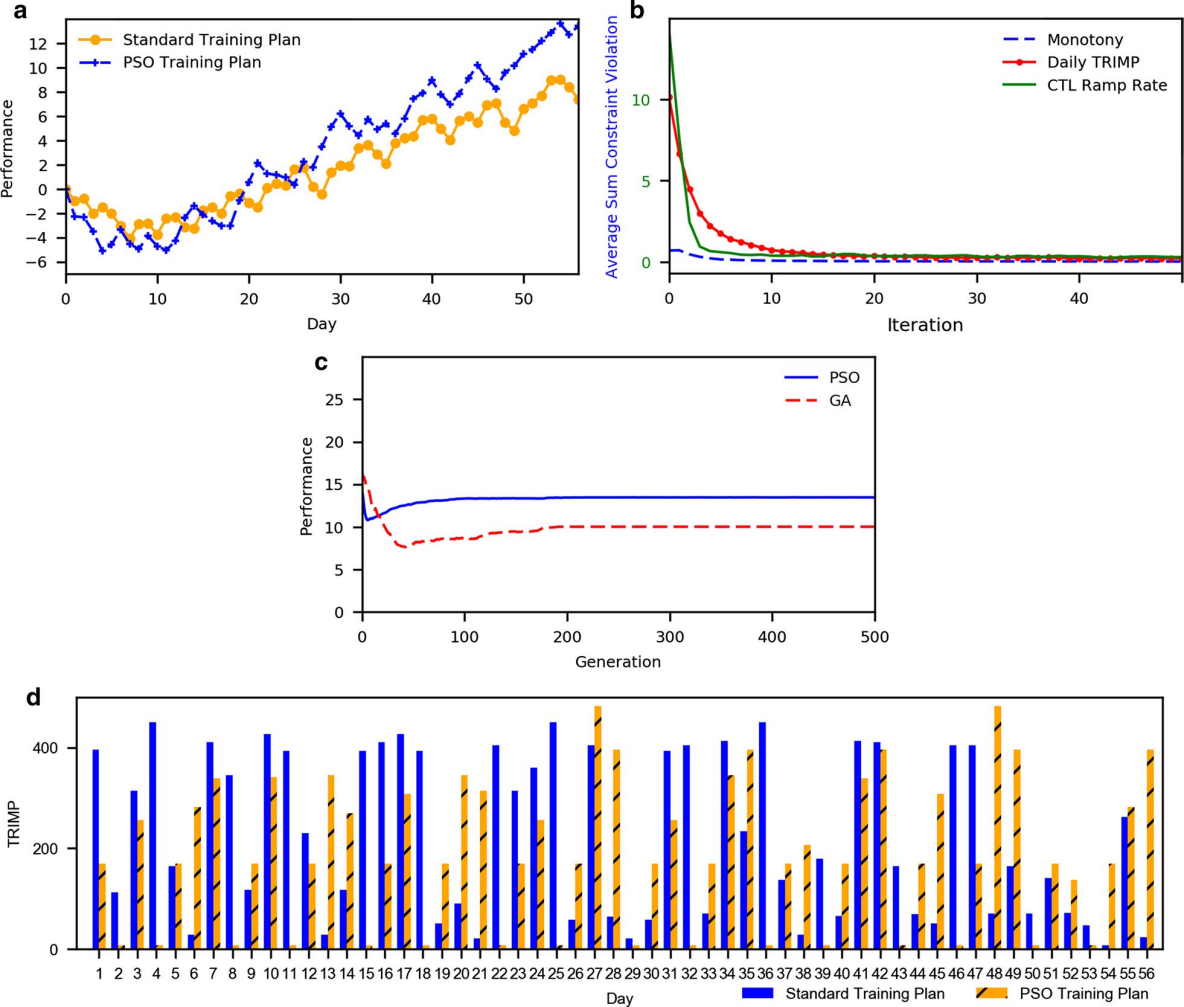
Summary of the PSO result. **a** The athletic performance by each training days corresponding to PSO’s training plans and British Cycling’s training plans (the training plan with higher athletic performance is preferred); **b** the convergence of each constraints violation value by algorithm iterations (the lower value of constraint violation is preferred); **c** the convergence of athletic performance value by algorithm iterations (the higher value of performance is preferred); **d** the comparison of daily training effort between PSO training plan and British Cycling’s training plan which presented by daily TRIMP value (higher TRIMP mean high training effort)

The comparison of the PSO training plan against our standard training plan, British Cycling’s training plan, is illustrated in Fig. 1d. The training load for each training sessions in such plans represented as a bar chart. The solid bars located at left-hand side belong to PSO result while the striped bar at right-hand side belonging our standard training load. As shown in Fig. 1d, both training plans share the same training pattern of alternation between high and low-intensity training. Thus, the dynamic time warping (DTW) analysis was done as a similarity analysis. We bound the measured Euclidean distance between two similar agents in the two training plans at the same position to 1 and the 2 training plans that furthest apart to 0, PSO distance from standard training plan at 0.804. Once it close to 1, it can be interpreted that PSO training plan is closely similar to our standard training plan in term of training patterns.

The athletic performance by training days in each training plans is represented as line chart in Fig. 1a. The dashed line with the cross marked belong to athletic performance by days in PSO training plan while the solid line with the dot marked belong to athletic performance by days in our standard’s training plan. Even though our results are closely related to the simulated standard training plan outcomes, the generated training plan outperformed the standard by raising athletic performance to a higher level of achievement. As shown in Fig. 1a, the PSO training plan performance is 13.436 while our standard training plan performance is 7.38. The PSO training plan was demonstrated to be a high-performance training plan which also satisfied all physiological constraints included.

### Constrained optimization

The performance of constrained optimization in this paper is illustrated in Fig. 1b which represented by line chart of sum constraints violation value of each constraint by iterations. The solid line, dashed-line and a dashed-dotted line are belonging to the monotony constraint, CTL ramp rate constraint and daily training load constraint respectively. The proposed technique also outperforms the GA-based technique [20] in terms of fast convergence and high quality solution, as illustrated in Fig. 1c. In addition, the performance of constrained optimization is analyzed in term of the sum of constraints violation by different iterations. In Table 2, we present statistic of the sum constraints violation including the best, the worse, an average and a standard deviation.

**Table 2:** All constraint violations in particular evaluation times (FEs)

FEs	Monotony constraint				CTL ramp rate constraint				Daily training load constraint			
	Best	Worst	Average	SD	Best	Worst	Average	SD	Best	Worst	Average	SD
50	1.452	2.908	1.531	0.183	0	9	0.227	1.220	0	24	0.307	1.966
500	1.507	2.531	1.522	0.106	0	10	0.163	1.153	0	19	0.22	1.739
50,000	1.507	2.953	1.522	0.134	0	10	0.093	0.843	0	20	0.186	1.681

Variation of a particle’s velocity by the algorithm’s iterations is fast in the early iterations (as shown in Fig. 1b) with a brief scanning of their nearby area. The particle’s velocity then slows down later as more detailed and fine searching occurs, seeking the best potential solution nearby their current position. The purpose of the adaptive maximum limit of the particle’s velocity is to avoid flying over better solutions. Table 2 illustrates the capability of this approach in each of iteration. Particles are able to find feasible solutions and attract others to move toward their positions. The best particles satisfying all constraints include monotony, CTL ramp rate and daily TRIMP restriction. This means that the adaptive PSO generated training plan is considered as a practical sports training plan that minimizes the risk of becoming overstrained.

## Conclusion

The adaptive PSO techniques for generating a sports training plan is presented. Since the problem domain in this paper is a cycling training plan, the training-performance model and cycling physiological constraints were adopted. This work included several processes including problem formulation, particle encoding, athletics performance model implementation as the objective function, physiological constraints adoption and implementation of an adaptive PSO with the *ɛ*-constraint method as the main optimization technique. Our simulations demonstrated that the PSO-generated training plan significantly outperformed the standard plan based on a training plan from British Cycling while satisfying all physiological constraints. We have demonstrated that the Adaptive Particle Swarm Optimization method of driving a training plan and considering certain physiological constraints, produces a safe, high-performance training plan. However, this training plan is needed some further tests and analyses with human participation to ensure its safety in practice.

## Limitations

For the best training result, parameters of training-performance interaction model need to be adjusted regarding particular athlete’s physical adaptation on particular sports.

Further, unplanned overtime jobs, family issues and illness may intervene in the athlete’s training plan. Reorganization of the training plan may be needed to cover these unpredictable issues to maintain or raise athlete’s performance as much as possible in the remaining time until the race day.

## Additional file

**Additional file 1.** Description of data: pseudocode of the proposed technique.

## Abbreviations

CTL: chronic training load
PSO: particle swarm optimization
HR: heart rate
bpm: beats per minute
D: duration
FTHR: Functional Threshold Heart Rate
TRIMP: Training IMPulse
DTW: dynamic time warping
