# Supplementary material for: Planning a sports training program using Adaptive Particle Swarm Optimization with emphasis on physiological constraints
Source: BMC Res Notes. 2018 Jan 8;11:9. doi: 10.1186/s13104-017-3120-9 (PMC5759209; doi:10.1186/s13104-017-3120-9)
Supplement: Supplementary file 1 — Additional file 1. Description of data: pseudocode of the proposed technique. [file 13104_2017_3120_MOESM1_ESM.docx]

$$\begin{matrix} \text{Randomly} \text{initiate} \text{particle's} \text{position},x_{i} \\ \text{Evaluate} f(x_{i}) \text{and} \text{particle's} \varphi(x_{i}) \\ \text{Update} \text{p}-\text{best's} \text{position}, x_{i}^{*} \\ \text{Update} \text{p}-\text{best's} \text{constrain} \text{vioation,} \varphi(x_{i}^{*}) \\ \text{Randomly} \text{particle's} \text{velocity} \text{vector.} \\ \text{Initialize} \text{maximum} \text{velocity},V_{\max} \\ while (not meet exit criteria)\{ \\ \text{Update} \text{particle's} \text{velocity}, v_{x_{i}}, \text{limiting} \text{to} V_{\max} \\ \text{Move} \text{particle's} \text{position} \text{regarding} \text{to} \text{its} \text{velocity} \text{vector.} \\ \text{Evaluate} \text{particle's} \text{objective} \text{value.} \\ \text{Evaluate} \text{particle's} \text{sum} \text{of} \text{constraints} \text{violation.} \\ \text{Initate} \text{particle's} \text{best,}x_{i}^{*}, \text{and} \text{global} \text{best}, x_{G} \\ if (f(x_{i})<_{\varepsilon}f(x_{i}^{*}))\{ \\ if (f(x_{i})<_{\varepsilon}f(x_{G}))\{ x{}_{G}=x_{i}\} \\ x_{i}^{*}=x_{i} \\ \} \\ \text{Update} V_{\max} \text{corresponding} \text{to} \text{current} \text{iteration.} \\ \} \end{matrix}$$

Pseudocode of the proposed technique
